# Editorial: New directions and trends in parenting research

**DOI:** 10.3389/fpsyg.2024.1476190

**Published:** 2024-08-16

**Authors:** Yosi Yaffe, Nicolette Vanessa Roman, Dorit Alt

**Affiliations:** ^1^Faculty of Education and Teaching, Tel-Hai Academic College, Qiryat Shemona, Israel; ^2^Centre for Interdisciplinary Studies of Children, Families and Society, University of the Western Cape, Cape Town, South Africa

**Keywords:** parenting, children, adolescents, new directions, research

For centuries, the study of child socialization has focused primarily on understanding the effects of traditional parenting styles, dimensions, and behaviors on the developmental trajectories and outcomes of children and adolescents. While these core issues remain highly significant, recent research in parenting aims to broaden our understanding by exploring contemporary aspects and fields within this domain. Parenting science has expanded significantly over the past five decades to understand parenting, the parent-child relationship, child development and the adjusted adult. The shift has been from behaviorism to attachment and child development, positive parenting, and the role of technology. More recently there has been an increased focus on father involvement, adverse childhood experiences (ACEs), genetics and epigenetics, the role of culture on parenting and evidence-based interventions providing more scientifically backed parenting programs and interventions. The findings and insights stem from the studies included in the current Research Topic resume those recent trends in parenting research and further enhance our knowledge of the numerous factors that influence parenting and its impact on children's and adolescents' development. Key areas of interest include cultural and gender significances in parent-child relationships, the importance of other socialization agents, parental mental health, unconventional or novel parenting patterns alongside traditional parental behaviors, parenting children with disabilities, and family dynamics that influences children's creative and academic outcomes. By exploring these exciting directions in parenting research, we can deepen our understanding of how various elements affect parenting practices and outcomes, ultimately contributing to the wellbeing of children and adolescents.

In this Research Topic, we intended to show new directions for parenting science. In particular, the study of human “capital” which is used in different ways by Hazan-Liran and Wang et al.. Hazan-Liran focuses on psychological capital which can influence how parents cope with challenges, their overall wellbeing, and their ability to provide effective parenting. Wang et al. symbolic capital can affect children's social standing among peers, access to resources, and overall wellbeing. A holistic overview of the studies in this Research Topic, show both overtly and covertly, the importance of focusing on mental health in and through the parent-child/adolescent relationship. We know that mental health plays a key role in the way parents and children interact and engage with each other, in the family and with the wider environment (see Egami). The new directions encourage a holistic understanding of the psychological, social, and organizational factors, within and outside of the family environment, that affect parenting, particularly for those facing unique challenges, such parents with children with a disability, thereby paving the way for innovative approaches and solutions in the field of parenting research. These new directions integrate social, cultural, and economic factors, for culturally relevant, and technology-driven approaches to provide parenting support, ultimately contributing to improved child health and family wellbeing in diverse contexts. Nguyen and Nguyen reported that conflicts often arise between Vietnamese parents and their teenagers over using the internet and schoolwork. Consistent with evidence from western families, in these families, adolescents indicated experiencing a higher frequency of conflicts with their mothers compared to their fathers. Interestingly, despite the identification of instances involving parental aggression, the majority of adolescents perceived their parents' approaches to conflict resolution as supportive. The findings highlight the importance of investigating parenting styles and parent-child communication within the cultural contexts such as modern Vietnamese families. Additionally, Chen et al. analyzed the relationship between parental psychological control and young children's peer interactions in Chinese families, while considering the moderating effect of teachers' emotional support in this context. They found that parental psychological control was negatively associated with young children's peer interactions, while teachers' emotional support moderated this relationship, mitigating the adverse effects of the parental control factor. The study findings offer valuable insights for integrating elements of the proximal system and developing interventions aimed at creating a harmonious home-school environment that promotes children's social development. Jin and Ahn tested the mediating role of maternal anxiety and the moderating role of mindfulness in the relationship between work–family conflict and preschool children's problem behaviors during the COVID-19 epidemic. Their findings indicated that maternal anxiety mediates the positive association between work–family conflict and children's problem behaviors, suggesting that children of mothers with work–family conflict exhibit more behavioral problems at least partially due to their mothers' increased anxiety. The study findings shed more light on the significance of parental mental health in explaining child's behavior, while signifying the potential role of mindfulness practices in restraining the former's negative effects.

Parenting behavior plays a crucial role in shaping children's development and academic success. The paper titled “*Associations between challenging parenting behavior and creative tendencies of children: the chain mediating roles of positive emotion and creative self-efficacy*” (Shi et al.) explores how parenting that encourages children to face and overcome challenges can foster their creativity. It delves into the mechanisms through which positive emotions and creative self-efficacy mediate this relationship, offering insights into how parental actions can influence a child's creative potential. This research is particularly relevant as it highlights the importance of fostering a positive emotional environment and nurturing a belief in one's creative abilities, which can significantly impact a child's creative output. Complementing this, the paper, “*Parent-adolescent discrepancies in educational expectations, relationship quality, and study engagement: a multi-informant study using response surface analysis*,” (Song et al.) examines the dynamics between parents and adolescents regarding educational expectations. It investigates how differences in these expectations can affect the quality of their relationship and the adolescent's engagement in their studies. This study provides a nuanced understanding of the complexities involved in parent-adolescent interactions and how mismatched expectations can influence academic engagement and relational quality. Hence, both papers provide valuable perspectives on how parenting and relational dynamics influence children's creative and academic outcomes. These insights can inform the development of educational programs that not only enhance knowledge but also foster positive emotional and relational environments, ultimately contributing to more effective learning and engagement. Additionally, they offer guidance on how parents and educators can balance challenges and expectations to cultivate creativity and maintain strong, supportive relationships, thereby improving educational experiences for children and adolescents. Furthermore, these studies highlight the need for targeted interventions that address specific parent-child dynamics, enhancing overall family wellbeing and academic success. Furthermore, these studies can inform policy-making by emphasizing the importance of parent education programs that teach effective communication and expectation management strategies. This could lead to improved family dynamics, and better educational outcomes for children.

